# Pneumothorax

**Published:** 1908-02-29

**Authors:** J. E. Squire

**Affiliations:** Senior Physician to the Mount Vernon Hospital for Consumption.


### Hospital Clinics.

### PNEUMOTHORAX.

By J. E. SQUIRE, C.B., M.D., Senior Physician to the Mount Vernon Hospital for Consumption.

(Abstract of a lecture delivered at the Medical Graduates' College and Polyclinic on February 13.)

The subject of which I have to speak to you this afternoon is that of air in the pleural sacs. Air may reach this situation in two ways: from the outside through an opening in the chest-wall, as, for example, by injury, stab or bullet wound, or the operation of paracentesis; and from the inside by perforation of the pleura covering the lung.

Curiously enough, wounds of the chest-wall very rarely result in pneumothorax. The opening is generally large enough for air to pass easily, and one might expect that it would enter the pleural sac. But the cohesion of the moist surfaces of the pleura is so strong that the air merely passes in and out of the wound with respiration, without separating one layer from the other.

The number of cases of pneumothorax from an external wound in a healthy chest-wall is extremely small: when, however, there is perforation of the pulmonary pleura, matters are different. At each inspiration air gets through the pleural opening, but during expiration it cannot return, owing to a valvular action of the perforated membrane. The next expiration further separates the chest-wall from the lung, and thus more and more air is imprisoned until the lung is collapsed and a pneumothorax established.

The chief cause of this condition is pulmonary tuberculosis. Over a focus of softening or cavitation the pleura is thinned and easily perforated. A sudden violent expiratory effort as a rule completes the rupture, and hence it is often a cough that determines the occurrence of pneumothorax. In a healthy lung a perforation is very unlikely from such a cause, for a pressure of from three to nine millimetres of mercury is required to rupture the normal lung. The usual estimate is that 90 per cent. of the cases of pneumothorax are due to pulmonary tuberculosis. The lesion occurs also in whooping-cough, broncho-pneumonia, and, it is said, sometimes in emphysema.

I have notes here of an interesting case which illustrates one of the rarer causes of pneumothorax, the bursting of an empyema into a bronchus. A bricklayer, of 43 years of age, had a history of cough, fœtid expectoration, and shortness of breath for nine months. When admitted to hospital he had impaired breath sounds at the right base with a wooden percussion note. I ought to mention that there were no tubercle bacilli in the sputum. Eight days afterwards he complained of pain in the chest, and dyspnœa and cyanosis developed: on examination it was found that the breath-sounds were feeble at the apex on the right side, absent altogether lower down; the bell sound, or bruit d'airain, was present. The man died about three days later.

Occasionally one comes across cases of pneumothorax in people whose lungs are presumed to be perfectly healthy. These are uncommon, and some people think there is always unsuspected tuberculosis existing. I saw such a case some years ago: a man who had had no illness except occasional slight bronchitis got sudden pain and dyspnœa while doing his usual morning physical exercises. He presented all the physical signs of pneumothorax. In the *British Medical Journal* for December 30, 1905, there is an account of a school teacher who sustained a pneumothorax while in his bath; there was no history of any previous lung illness whatever, and although succussion splash was obtained he got quite well in five weeks. The lungs were examined two years later, and were then apparently perfectly healthy.

I have said that 90 per cent. of the cases of pneumothorax are due to tuberculosis. What proportion of sufferers from this disease develop pneumothorax? On this point there is a very wide diversity of opinion in the literature. Fussell and Riesman in 58,731 cases of tuberculosis found 433 of pneumothorax, a proportion something under 1 per cent. At the Brompton Hospital in 1,000 cases they find a proportion of 6.5 per cent., a great difference from the German series. I have gone through the case-books for the last six or seven years at the Mount Vernon Hospital, and the proportion in 3,786 cases of phthisis there is only .5 per cent. Moreover, I went a stage further, and among 1,000 cases from our branch hospital at Northwood, whither are taken chiefly those in the early stages of the disease, whom we hope to cure, I find but one instance of pneumothorax. From these figures I am led to think that the differences of opinion about this matter are due to the nature of the cases dealt with. Pneumothorax is an accident of the advanced, rather than of the early stages of consumption, though cavitation is not necessary for its production.

### Symptoms.

What are the symptoms of pneumothorax? The accident as a rule occurs suddenly, and we should, therefore, expect a sudden onset of symptoms. In the ordinary way this is so: as a result either of cough or of unusual movement, the patient experiences a sharp and sudden pain which is not only acute, but conveys also the sensation of something having given way in the chest. A fear of impending death, of the kind often called cardiac anxiety, and shortness of breath at once follow. The patient is often unable to stand, and faintness and giddiness sometimes attack him. The dyspnœa increases, though the pain loses in intensity, and the case soon resolves itself into a fight for breath. In this state the patient may die without ever showing any signs of recovery; or things may settle down as the shortness of breath gradually diminishes. In a few days or weeks the patient may have completely recovered, though unfortunately this is unusual.

Such is the common mode of onset. The condition may, however, supervene gradually. Thus patients sometimes wake up with an increasing dyspnœa, which may not become intense for a considerable time. In either case, when the condition eases down it may be several months before all air is removed from the pleural cavity.

### Physical Signs.

The first point to bear in mind is that the physical signs do not come on immediately with the advent of the sharp pain, but become gradually more and more distinctive for several days. Even when from the symptoms you have a strong suspicion of what has happened, you may not at first find the physical signs of pneumothorax, though they may develop later. Here is a case in point. A patient who complains of pain in the chest is found to have pleural friction on July 7. On the 11th he complains also of dyspnœa, but his physical signs remain unaltered. By the 14th he has clear signs of pneumothorax, and two days later he dies suddenly.

The physical signs, then, are these. The chest is fixed on the affected side, and the intercostal spaces may be obliterated, as in effusion; but the percussion note is hyper-resonant. The breath-sounds are absent, or very feeble. The bell sound is to be obtained, though it should be remembered that a large cavity close under the pleura may cause the same result. After two days fluid will probably be poured out, and you may then get succussion splash, or that other characteristic sign, the metallic drip of fluid falling into other fluid in a large resonating chamber. As a rule there is considerable displacement of organs: with pneumothorax on the left side there may be a cardiac apex beat near the right nipple. I have quite recently been able to show a case of this sort at Hampstead. A labourer, 24 years old, has had cough and expectoration in which tubercle bacilli were found on January 1 this year. On the 7th he had sudden severe pain on the left side of the chest, and developed the signs of pneumothorax. The cardiac dulness was wholly to the right of the sternum, and the apex beat was one inch internal to and below the right nipple. Since then the heart has come back a little way, but the apex is still to the right of the sternum. Curiously enough, this man has never had any very marked dyspnœa. This symptom depends very largely on the rapidity with which the position of the heart is altered. When the process occurs slowly, accommodation to the altered conditions is much more easily effected.

In this particular case there was a sudden fall of temperature on the evening before the perforation occurred. Besides this event, which is sometimes noticed, a very constant increase of pulse and respiration rate takes place, in this patient from 92 to 120 for the former, and from 24 to 36 for the latter.

If you examine the chest with the $x$-rays you obtain a very different view from that afforded by pleural effusion. The lung may be found compressed into an extremely narrow wedge which gives a slight shadow near the root of the viscus.

### Prognosis.

The result of pneumothorax is frequently fatal: something like 70 to 75 per cent. die, and only about 10 per cent. ever recover completely. According to West, about 50 per cent. of the patients die during the first week of the illness. The first danger is that of cardiac failure from the sudden displacement of the heart. After that death from dyspnœa, suffocation, and exhaustion may occur in the first twelve to twenty-four hours, in which the greatest peril is. When fluid is poured out there are additional dangers, especially if that fluid be purulent. In almost every case a hydropneumothorax forms, and when it is a pulmonary cavity which has burst there is nearly always a pyopneumothorax afterwards.

### Treatment.

During the urgent dyspnœa it may be necessary to tap the chest and relieve the symptoms by removing the source of pressure. Paracentesis should be performed in all cases of great cardiac embarrassment. Suction must not be employed, and, therefore, an aspirator is not needed, but a cannula should be used, connected with a long tube the lower end of which dips into water. This tapping may be repeated several times if necessary. There was an account published a little time ago of a case in which paracentesis was done with the guide of an $x$-ray screen. There seems to be some advantage in this, for it can be seen whether the lung expands, and the cannula can be withdrawn so as not to prick it. This method of course cannot be used in private practice in a sudden emergency; but when it can be adopted it seems useful.

Apart from the removal of air to relieve acute symptoms, prop the patient up and give him stimulants to keep up his strength, such as ether, ammonia, strychnine, etc. If pain and anxiety are acute opium or morphine may be necessary. When fluid begins to accumulate, act as if there was no air present. Aspirate, and if you draw off pus, operate as for any other empyema. There is, as you know, a view taught that compression of the lung by fluid hinders or checks tuberculous processes in it, but I do not share that impression. The same applies to compression by air, and personally I am not in favour of leaving the lung collapsed, whether it is by air, serum, or pus.

There remain two or three cases the notes of which I have brought to illustrate certain points. Thus a postman, aged 26, had pulmonary tuberculosis for two years. There was extensive mischief on both sides, but he was doing well, gaining weight, and generally improving, when one night he felt sick. Next morning the temperature fell quickly, and he vomited and complained of headache. Already the physical signs were very suggestive of pneumothorax. Four days later the headache was worse, and the patient became drowsy and cyanotic, with physical signs of pneumothorax well marked. Ten days after the first onset of these symptoms he died. Here is a case in which there was no sudden pain, unless the cerebral condition was sufficient reason for no complaint of it to be made. Whether the vomiting was a sign of the development of pneumothorax, or whether the latter was caused by the strain of the former is uncertain. The fall of temperature, which appeared before the vomiting, suggests the former alternative.

Another case is that of a woman of 21 who had been suffering from tuberculosis of the lungs for twelve months. It may be remarked here that pneumothorax is four times as common in men as in women. She was getting on fairly well, when suddenly she felt intense pain in the right breast; a friction rub was heard, but no signs of any condition other than pleurisy could be detected. Two days later the breath sounds were still heard, though more faintly, but on the following day there were all the indications of a pneumothorax. In this case the respiration-rate ascended to over 50 per minute directly the pneumothorax was established.

Another woman, aged 27, developed sudden severe dyspnœa one month after her admission to hospital for pulmonary tuberculosis. The physical signs were very much as they had been before, and next morning she seemed much better, though her pulse-rate was still as much as 120. Not until four days later did she present the signs characteristic of pneumothorax.

I am afraid I have not been able to tell you anything very new, but I hope I have put matters sufficiently plainly to remind you of the signs of this trouble, and to show you that the accident is not such a very rare one to meet with in hospital practice.

